# Synergistic Pathogenic Effects of *Riemerella anatipestifer* and *Infectious Bronchitis Virus* in Layer Chicks

**DOI:** 10.3390/ani16152294

**Published:** 2026-07-23

**Authors:** Xiaoman Bai, Jianhao Chen, Jie Zhu, Yantao Wu, Xiaorong Zhang

**Affiliations:** 1 Jiangsu Co-Innovation Center for the Prevention and Control of Animal Infectious Disease and Zoonoses, College of Veterinary Medicine, Yangzhou University, Yangzhou 225009, China; mx120241047@stu.yzu.edu.cn; 2 Jiangsu Hai’an Layer Science and Technology Backyard, Jiangsu Kangde Egg Industry Co., Ltd., Nantong 225009, China; chenjianhao@jskangde.com; 3 Shandong Binzhou Wohua Bioengineering Co., Ltd., Binzhou 256606, China; zhujievet@163.com; 4 The International Joint Research Laboratory of Agricultural and Agri-Product Safety, Yangzhou University, Yangzhou 225009, China

**Keywords:** *Riemerella anatipestifer*, chicken, *infectious bronchitis virus*, layer chicks, oviductal obstruction, synergistic pathogenicity

## Abstract

In recent years, *Riemerella anatipestifer* (RA) has been detected with increasing frequency in layer chick flocks. RA infection can lead to reproductive tract lesions, including salpingitis. Epidemiological surveillance further indicates that RA infection often occurs as a co-infection with other respiratory pathogens, among which *infectious bronchitis virus* (IBV) is the most common. To elucidate the pathogenicity of chicken-derived RA and its synergistic effects with IBV, a serotype 10 RA strain and a GI-19 lineage IBV strain co-isolated from a layer chick with oviductal obstruction were used in animal experiments to compare disease outcomes following single infection and co-infection in layer chicks. The results demonstrated that IBV single infection caused typical clinical signs and pathological lesions characteristic of infectious bronchitis, but did not induce pericarditis, airsacculitis, or oviductal obstruction. By contrast, aerosol challenge with chicken-derived RA resulted in pronounced pericarditis, airsacculitis, and caseous salpingitis. Further co-infection experiments showed that IBV combined with chicken-derived RA exacerbated clinical signs and pathological lesions, suggesting a synergistic interaction between the two pathogens. This study confirms chicken-derived RA as a major primary pathogen in caseous oviductal obstruction of layer chicks, and demonstrates its synergistic pathogenic effects during IBV co-infection. These findings support the etiological diagnosis and targeted prevention and control of oviductal obstruction in layer chicks.

## Simple Summary

This study investigated the unclear etiology of oviductal obstruction in layer chicks and the poorly characterized pathogenic role of co-infection with *Riemerella anatipestifer* and *infectious bronchitis virus*. The objective was to characterize the pathogenicity of *Riemerella anatipestifer* and evaluate its synergistic effects during *infectious bronchitis virus* co-infection. The results showed that *infectious bronchitis virus* single infection primarily induced typical bronchitic lesions but did not cause pericarditis, airsacculitis, or oviductal obstruction. In contrast, aerosol challenge with chicken-origin *Riemerella anatipestifer* alone induced marked pericarditis, airsacculitis, and caseous oophoritis. Co-infection with both pathogens further exacerbated clinical signs and pathological lesions. These findings suggest that *Riemerella anatipestifer* is a major primary pathogen associated with caseous oophoritis in layer chicks, and that its pathogenicity is enhanced by *infectious bronchitis virus* co-infection. This study provides a scientific basis for etiological diagnosis, targeted prevention and control strategies, and the reduction of economic losses in poultry production.

## 1. Introduction

*Riemerella anatipestifer* (RA) is a Gram-negative, rod-shaped bacterium classified within the genus Riemerella, family Flavobacteriaceae, and phylum Bacteroidetes [1]. It was first isolated and identified from diseased ducks in the United States by Hendrickson, Hilbert, and colleagues in 1933 [2]. Subsequently, RA infections were reported worldwide, and the bacterium became widely distributed, emerging as a major pathogen of the global waterfowl industry. In 2021, RA infection was documented in commercial broiler chickens in Greece (Ross 308 breed) [3]. Although RA primarily affects ducks and turkeys, sporadic cases and outbreaks in chickens have also been reported in different regions of China [3,4,5]. RA is now recognized as a major bacterial pathogen threatening the global waterfowl industry, capable of causing acute or chronic septicemia and polyserositis in various poultry species and leading to substantial economic losses [6]. In ducks, typical pathological lesions associated with RA infection include fibrinous pericarditis, airsacculitis, perihepatitis, and caseous salpingitis [7]. In recent years, the host range of RA has been expanded, and RA has been gradually transformed from a traditional waterfowl pathogen into an important bacterial pathogen affecting chicken flocks. RA is now widely prevalent in broiler, layer, and breeder chicken flocks [4], causing substantial economic losses to the poultry industry [8,9,10]. Tissue-specific detection of RA was performed in dead or culled layer chicks that exhibited typical clinical signs of RA infection. The results showed that the detection rate of RA in oviduct tissues exceeded 20% [5]. In layer chick production, the oviduct is a critical component of the reproductive system, and its structural and functional integrity directly affects production efficiency. Oviductal inflammation caused by RA infection can lead to decreased egg production, reduced egg quality, and a shortened layer chick cycle [11]. Given its high prevalence and wide-ranging impact in layer chick flocks, RA is recognized as one of the major bacterial pathogens constraining the economic efficiency of egg production [12].

RA exhibits substantial serotype diversity, with at least 25 serotypes reported worldwide to date. Strains of different serotypes differ markedly in pathogenicity [13], and the lack of cross-protection among serotypes poses major challenges for vaccine development and clinical prevention and control [14]. In China, RA serotypes 1, 2, and 10 are the predominant circulating serotypes [15]. Among these, RA serotype 10 has been detected at a high frequency in layer chicks and broiler breeders in China. This serotype can induce typical serositis and oviductal lesions, resulting in increased mortality and a marked decline in growth performance [5]. Furthermore, as poultry production has intensified, new serotypes and genotypes of chicken-derived RA isolates have been reported, making control efforts increasingly difficult and underscoring the need for pathogenicity studies of predominant serotypes.

Zhang et al. [5] carried out a nationwide cross-sectional epidemiological survey of 5473 commercial chicken farms distributed across 29 Chinese provinces from 2021 to 2024. Standardized tissue sampling, bacterial isolation and species-specific PCR were adopted to identify *Riemerella anatipestifer* (RA), and multiplex PCR was further utilized to screen co-infecting pathogens of the respiratory and reproductive tracts. Weighted statistical analysis of 27,136 clinically diseased samples indicated that merely 20.18% of RA-positive laying hen cases were single RA infections. Instead, RA infections in field chickens were dominated by mixed infections with multiple avian pathogens, among which the co-infection proportion of infectious bronchitis virus (IBV) reached 46.93%. IBV belongs to the genus Gammacoronavirus within the family Coronaviridae. It is an acute, highly contagious pathogen that primarily infects epithelial tissues in the respiratory tract, kidneys, and reproductive tract of poultry [16], thereby triggering local and systemic immune responses. To date, global IBV strains exhibit extensive genotypic diversity, with the GI-19 lineage accounting for the majority of isolates recovered from poultry-rearing countries worldwide [17,18,19]. IBV infection predisposes birds to secondary infections with other pathogens, thereby exacerbating disease severity [20]. A substantial body of research has described the synergistic pathogenicity between respiratory viruses and bacteria, including the synergistic effects of low-pathogenicity chicken-derived influenza virus with chicken-derived *Escherichia coli* and chicken-derived reovirus with *Staphylococcus aureus*. The underlying mechanisms are often associated with virus-induced disruption of the epithelial barrier and suppression of host immune function [21,22]. However, systematic experimental validation of the interaction mechanisms between RA and IBV, particularly their synergistic pathogenic effects in layer chicks, remains limited. In clinical settings, oviduct impaction in layer chicks is frequently associated with co-infections; however, the primary etiological agent remains largely undetermined. Current research primarily focuses on the pathogenicity of RA single infection or the management of IBV single infection. In contrast, studies detailing the clinical epidemiology, pathological characteristics, pathogenic mechanisms, and pathogen interactions during RA and IBV co-infection remain scarce. This knowledge gap deprives clinical diagnosis and disease management of a targeted theoretical foundation, thereby hindering the implementation of precise control strategies in poultry production and ultimately exacerbating economic losses.

This study investigated a chicken-derived RA serotype 10 strain and an IBV strain belonging to the GI-19 lineage, both isolated from a clinically affected layer chick with oviduct impaction. Using animal models, this study evaluated the differential pathogenicity of these pathogens in layer chicks following single infection or co-infection. Furthermore, it explored their synergistic pathogenic effects, aiming to provide a theoretical basis and empirical support for the comprehensive prevention and control of RA and IBV co-infections in clinical settings.

## 2. Materials and Methods

### 2.1. Bacterial Strains and Viruses

The serotype 10 chicken-derived RA strain G827 and the GI-19 lineage IBV strain D65 were previously co-isolated and identified in November 2024 from a diseased chicken exhibiting oviduct obstruction. To ensure bacterial viability, the RA strain G827 was passaged twice on tryptic soy agar (TSA) supplemented with 5% fetal bovine serum (FBS). The bacterial isolate was subsequently confirmed via the observation of colony morphology, Gram staining, and specific PCR amplification of the 16S rRNA gene. Universal primers (F: 5′-CAGCTTAACGTAGAACTGC-3′; R: 5′-TCGAGATTTGCATCACTTCG-3′) yielding an expected 662-bp amplicon were synthesized by GenScript Biotech (Nanjing, China) according to a previous study [23]. The IBV strain D65 was revived by passage in embryonated chicken eggs, and its viral titer was determined to be 10^4.5^EID_50_/0.2 mL. The identity of the virus was further verified by S1 gene sequencing. The virus stock was subsequently aliquoted and stored at −80 °C until further use.

### 2.2. Animals

One hundred twenty healthy 1-day-old Hy-Line Gray layer chicks were purchased from Haian Tianfuyuan High-Quality Chicken Breeding Co., Ltd (Nangtong, China). The chicks were housed in individual cages within a negative-pressure isolator located in a biosafety level 2 (BSL-2) facility and provided with a 7-day acclimatization period before the experiment was initiated. All animal experimental procedures were conducted in accordance with the ARRIVE guidelines 2.0 and approved by the Animal Welfare and Ethics Review Committee of Yangzhou University (Ethics Approval No. 202503049).

### 2.3. Main Reagents

Tryptic soy broth (TSB) and tryptic soy agar (TSA) were purchased from Qingdao Haibo Biotechnology Co., Ltd. (Qingdao, China), and fetal bovine serum was purchased from Thermo Fisher Scientific Inc. (Shanghai, China).

### 2.4. Pathogenicity Test for Single Infection

As detailed in Table 1, sixty 14-day-old Hy-Line Gray layer chicks were randomly assigned to three groups (*n* = 20 per group). To simulate respiratory exposure to RA, birds were challenged by aerosolization in a negative-pressure isolator (3.5 m^3^) maintained at 28–30 °C and 50–70% relative humidity. For each challenge, 20 mL of bacterial suspension containing 1 × 10^9^ CFU/mL of RA strain G827 was loaded into a sterile pneumatic airbrush system fitted with a 0.3-mm nozzle and operated at 20 PSI. The suspension was aerosolized for 20 min, with the sprayer positioned approximately 30 cm from the birds. Based on the nozzle specifications and operating pressure, the generated droplets were estimated to be predominantly 10–50 μm in diameter. Ventilation was turned off during aerosolization and restored 20 min after the end of spraying, resulting in a total exposure period of 40 min. Assuming uniform distribution within the isolator, the total aerosolized bacterial load (2 × 10^10^ CFU) corresponded to a theoretical environmental concentration of approximately 5.7 × 10^6^ CFU/L of air. Because a whole-body aerosol exposure model was used, the exact inhaled dose for each individual chick could not be directly measured.

For oculonasal inoculation (Groups D2 and D3), each chick was manually restrained, and 0.2 mL of inoculum was administered dropwise into both nares and eyes using a micropipette; care was taken to avoid aspiration. At 14 days of age, chicks in Group D1 were inoculated with RA G827 bacterial suspension by aerosolization as described; chicks in Group D2 were inoculated with 0.2 mL of IBV D65 containing 10^4.5^EID_50_ via the oculonasal route; and chicks in Group D3 (negative control) received 0.2 mL of sterile PBS via the oculonasal route. On days 7, 14, and 21 post-inoculation, five chickens were randomly selected from each group and euthanized by cervical dislocation for examination of gross pathological changes in tissues and organs. All procedures were performed under sterile conditions in a biosafety level 2 facility.

### 2.5. Pathogenicity Test for Co-Infection

As shown in Table 2, sixty Hy-Line Gray layer chicks were used in this experiment, comprising two age cohorts: thirty 7-day-old birds and thirty 14-day-old birds. Within each age cohort, the birds were randomly assigned to three groups (10 birds per group): Group H1 (RA single infection), Group H2 (IBV and RA co-infection), and Group H3 (negative control).

**Figure 1:**
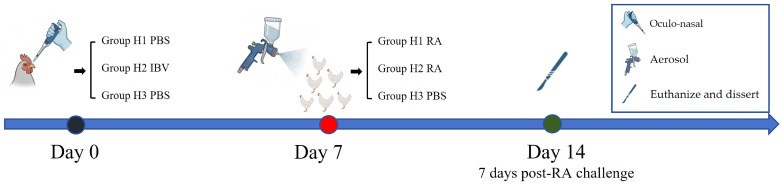
Schematic representation of the experimental design.

The experimental procedure was conducted chronologically as follows (Figure 1): 

On Day 0, birds in Group H2 (both 7- and 14-day-old) were initially inoculated with 0.2 mL of the IBV D65 strain (10^4.5^EID_50_) via the oculo-nasal route. Simultaneously, birds in Groups H1 and H3 were inoculated with 0.2 mL of PBS via the same route to serve as controls for this stage.

On Day 7, birds in Groups H1 and H2 were challenged with 1 × 10^9^ CFU/mL of RA G827 via aerosolization, while birds in Group H3 received an equal volume of PBS aerosol.

On Day 14 (seven days post RA challenge), all birds were euthanized, and Gross pathological lesions changes in tissues and organs were examined.

The detailed experimental design is illustrated in Figure 1.

### 2.6. Clinical Signs

After being challenged, the chickens were observed twice a day. Detailed records were maintained for mental status, feed and water intake, and various physical abnormalities, including respiratory signs (coughing, sneezing, and rales), neurological symptoms (ataxia and convulsions), and mobility issues (limping and paralysis). Additionally, the time of onset, the number of affected birds, and the number of deaths were recorded.

### 2.7. Assessment of Tracheal Ciliostasis

Five chickens from each group were sacrificed at 5 days post-infection and their trachea were examined since IBV causes serious damage to tracheal cilia. Tracheal rings were isolated from the upper (*n* = 3), middle (*n* = 4), and lower (*n* = 3) segments of the trachea and placed in DMEM containing 10% FBS. Ciliary motility was examined under a microscope at 400× magnification to determine the extent of ciliary movement and cilial loss. Ciliary activity was scored using a 0–4-point scale as follows [24]: 0, no damage and normal ciliary activity; 1, 75–100% ciliary activity; 2, 50–75% ciliary activity; 3, 25–50% ciliary activity; and 4, <25% ciliary activity or complete cessation of tracheal ciliary movement.

### 2.8. Gross Lesions

For the single-infection trials, five chickens from each group were randomly selected for necropsy at 7, 14, and 21 days post-infection. Conversely, in the co-infection trials, all chickens per group were necropsied seven days following the RA infection. During systematic dissection, target organs—including the pericardium, air sacs, oviduct, and liver—were examined for gross pathological lesions. Specific focus was directed toward fibrinous exudation in the pericardium, air sac thickening and opacity, caseous plugs and mucosal lesions in the oviduct, and inflammatory exudation in the liver. Representative macroscopic lesions were photographically documented.

### 2.9. Statistical Analysis

All experimental data were analyzed using GraphPad Prism software version 9.5. Categorical data, such as rates of pathological lesions and mortality, were expressed as percentages. Intergroup comparisons were performed using the chi-squared test or Fisher’s exact test, as appropriate. Continuous data were expressed as the mean ± standard deviation (SD). Comparisons among multiple groups were performed using one-way analysis of variance (one-way ANOVA). A *p* value of <0.05 was considered statistically significant, and a *p* value of <0.01 was considered extremely significant.

## 3. Results

### 3.1. Identification of RA and IBV

**Figure 2:**
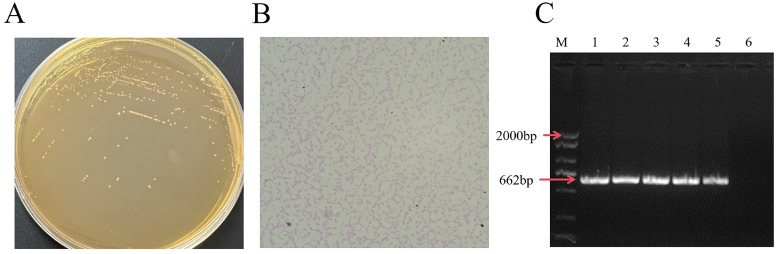
Morphological characteristics of the isolated RA G827 strain. (**A**) Colony morphology of the RA isolate on TSA; (**B**) Gram staining of the isolate (1000×); (**C**) Lanes 1–5: RA G827 strain; Lane 6: negative control.

On TSA plates with 5% FBS, the isolate produced typical milky white, slightly transparent, and convex smooth colonies (0.5–1.5 mm) (Figure 2A). Gram staining showed predominantly single Gram-negative short rods with occasional filamentous forms (Figure 2B). As shown in Figure 2C, PCR amplification using the DNA of strain RA G827 as a template yielded a specific amplicon corresponding to the expected target size (662 bp).

Regarding the IBV strain D65, following passage in embryonated chicken eggs, the viral titer was determined to be 10^4.5^EID_50_/0.2 mL. Furthermore, S1 gene sequencing and subsequent sequence analysis confirmed that the D65 isolate belongs to the GI-19 lineage.

### 3.2. Manifestations After Single Infection

#### 3.2.1. Clinical Observations

Throughout the observation period following the challenge, birds in all experimental groups (D1, D2, and D3) were monitored twice daily, and no obvious clinical signs were observed. The chickens maintained normal mental status, with both feed and water intake remaining stable and comparable across the challenged and negative control groups. None of the birds exhibited any respiratory signs, such as coughing, sneezing, or rales. Additionally, no neurological symptoms (e.g., ataxia or convulsions) or mobility issues (e.g., limping or paralysis) were recorded in any of the chicks. Furthermore, no onset of disease or mortality occurred in any group during the entire experimental period.

#### 3.2.2. Tracheal Ciliary Stasis Assessment

**Figure 3:**
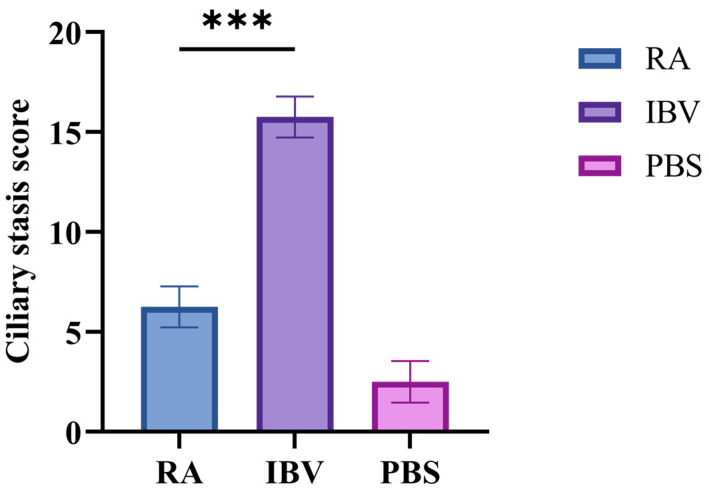
Ciliary stasis score. Data are presented as mean ± SD (*n* = 4). Data were analyzed using one-way analysis of variance (one-way ANOVA). *** *p* < 0.001.

At 5 days post-infection (dpi), examination of tracheal tissues revealed a marked accumulation of mucus in Group D2 (IBV group), accompanied by ciliary stasis and severe loss of cilia. Conversely, tracheal ciliary motility in Group D1 (RA group) and Group D3 (PBS control) remained unimpaired. Consequently, ciliary stasis scores were significantly higher in the IBV group compared to both the RA and control groups (Figure 3).

#### 3.2.3. Gross Pathological Lesions of the Pericardium, Air Sacs, and Oviduct

**Figure 4:**
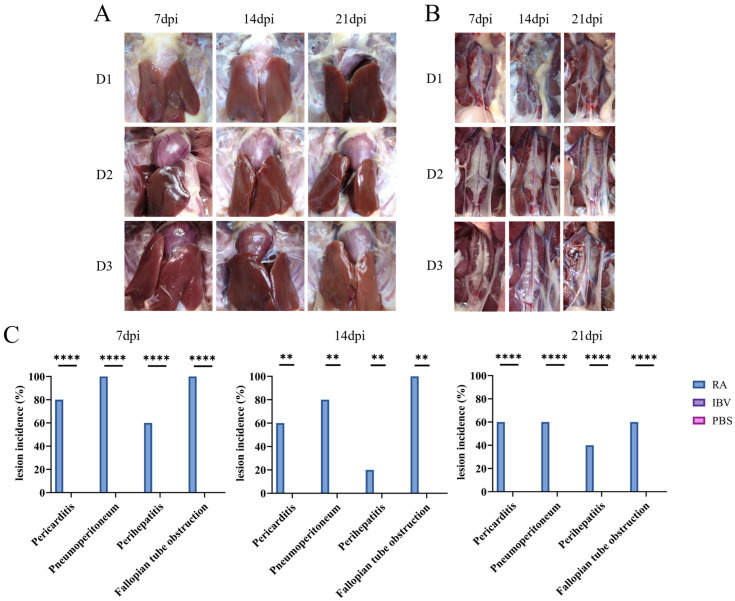
Gross pathological findings in layer chicks from different groups after challenge. (**A**) Pericardium and air sacs; (**B**) oviduct; (**C**) positive rates of gross pathological findings at different time points. Data were analyzed using GraphPad Prism 9.5 by one-way ANOVA. Significant comparisons are indicated in the figure by brackets and asterisks (** *p* < 0.01; **** *p* < 0.0001).

The remaining chickens in each group were necropsied at 7, 14, and 21 days post-infection (dpi) to evaluate gross tissue lesions. Throughout this observation period, varying degrees of fibrinous pericarditis, airsacculitis, perihepatitis, and oviductal occlusion were observed in the D1 (RA) group, whereas no discernible gross lesions were detected in either the IBV-only or blank control groups. As detailed in Table 3, the RA group exhibited a 100% incidence of both airsacculitis and oviductal occlusion (5/5) at 7 dpi, alongside an 80% incidence of pericarditis (4/5) and a 60% incidence of perihepatitis (3/5). At 14 dpi, the incidence of oviductal occlusion remained at 100%, while the rates of airsacculitis, pericarditis, and perihepatitis declined to 80%, 60%, and 20%, respectively. By 21 dpi, the incidences of airsacculitis, pericarditis, and perihepatitis each leveled at 60% (3/5). Representative macroscopic lesions are illustrated in Figure 4A,B, with the temporal dynamics of lesion-positive rates presented in Figure 4C.

### 3.3. Manifestations After Co-Infection

#### 3.3.1. Clinical Observations

In the co-infection pathogenicity trial, following RA challenge in 7-day-old chicks from Group H1 (RA single infected group), only 1 of 10 birds exhibited lethargy, with no observed dyspnea or mortality. When challenged at 14 days of age, these chicks displayed no obvious clinical signs or mortality. These findings indicate that RA single infection exerts relatively low clinical pathogenicity in chicks, primarily manifesting as mild clinical signs.

Conversely, in Group H2 (IBV and RA co-infection group), RA challenge in 7-day-old chicks resulted in 4 of 10 birds exhibiting typical respiratory signs, including depression and dyspnea, alongside a 30% mortality rate. Among chicks challenged at 14 days of age, 2 of 10 exhibited depression and dyspnea, with a 20% mortality rate.

Compared to the RA single infected group, clinical pathogenicity was markedly elevated in the co-infection group, as evidenced by more severe respiratory signs and higher mortality, particularly in younger birds. Overall, IBV and RA appear to exert a synergistic pathogenic effect, with younger birds demonstrating greater susceptibility.

#### 3.3.2. Postmortem Pathological Findings

As shown in Table 4, at 7 days post-RA infection, the 7-day-old H2 group experienced a 30% mortality rate (3/10). Among the seven surviving birds, the incidences of pericarditis, airsacculitis, perihepatitis, and oviductal occlusion were 85.71% (6/7), 57.14% (4/7), 85.71% (6/7), and 57.14% (4/7), respectively. In contrast, the RA single infection group exhibited no mortality, with lower incidences of pericarditis (50%, 5/10), airsacculitis (20%, 2/10), perihepatitis (50%, 5/10), and oviductal occlusion (20%, 2/10).

**Figure 5:**
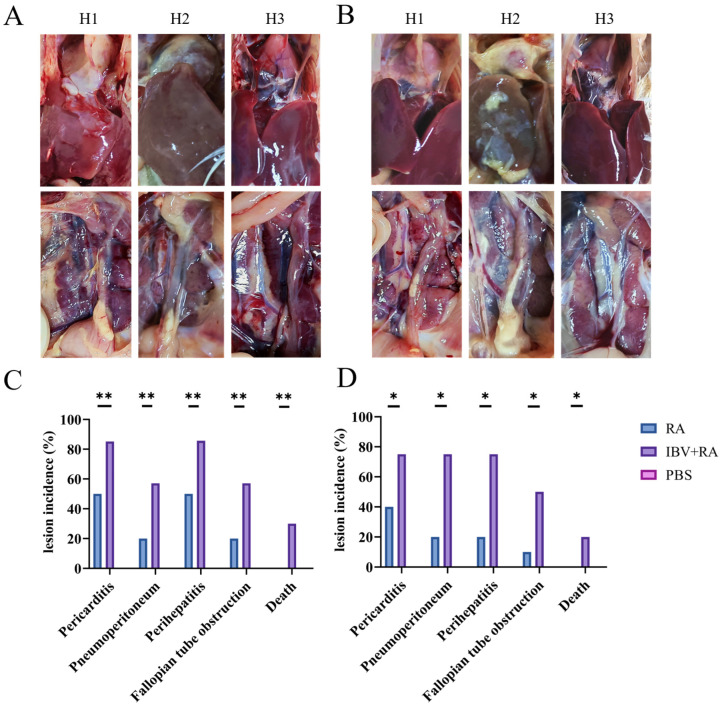
Gross pathological findings in the pericardial sac and oviduct of chicks at different ages after viral challenge. (**A**) 7-day-old chicks; (**B**) 14-day-old chicks; (**C**) positive rate of pathological indicators in 7-day-old chicks after co-infection; (**D**) positive rate of pathological indicators in 14-day-old chicks after co-infection. Data were analyzed using GraphPad Prism 9.5 by one-way ANOVA. ** *p* < 0.01; * *p* < 0.05.

Similarly, in the 14-day-old cohort at 7 days post-RA infection, the H2 group recorded a 20% mortality rate (2/10). Among the eight survivors, the incidences of pericarditis, airsacculitis, perihepatitis, and oviductal occlusion were 75% (6/8), 75% (6/8), 75% (6/8), and 50% (4/8), respectively. Conversely, the RA single infection group recorded zero mortality, with lesion incidences of 40% (4/10), 20% (2/10), 20% (2/10), and 10% (1/10) for the respective pathological indicators. Overall, the IBV and RA co-infected groups demonstrated significantly higher mortality and lesion-positive rates across all indicators compared to the RA-only groups (*p* < 0.05, chi-square or Fisher’s exact test). A summary of the pathogenicity results for the co-infection is presented in Figure 5.

## 4. Discussion

The mechanisms underlying bacterial–viral synergistic pathogenesis in poultry are highly complex. Disease severity and progression are governed by multiple factors, including inter-pathogen dynamics, pathogen virulence, host immune status, and environmental conditions. In this study, two in vivo experiments were conducted to systematically evaluate the pathogenicity of a primary infection with chicken-derived RA serotype 10, as well as a co-infection with IBV in layer chicks. Ultimately, this approach aims to elucidate the primary pathogenic role of this RA strain and the potential aggravating effect associated with IBV co-infection.

Results from the single-infection trials demonstrated that RA single infection elicited characteristic caseous salpingitis. Furthermore, at 7 days post-infection, the incidences of pericarditis and airsacculitis reached 80% and 100%, respectively. These findings indicate that chicken-derived RA is the primary pathogen responsible for pericarditis, airsacculitis, perihepatitis, and caseous salpingitis in layer chicks, corroborating previous reports [12,25,26]; In contrast, IBV single infection did not induce macroscopic oviductal lesions in the present study, a finding that contrasts with previous reports indicating that IBV can cause severe damage to the reproductive system. This discrepancy is highly likely due to the age of the birds at the time of infection. Previous studies have demonstrated that the timing of infection is critical, with typical IBV induced oviduct lesions (such as cystic oviducts) predominantly occurring when chicks are infected at day-old or within the first week of life [27]. In this study, the chicks were inoculated at 14 days of age, which may be too late a developmental stage for IBV alone to induce observable macroscopic lesions in the oviduct. Nevertheless, co-infection at this age exacerbated a potential interaction between IBV and RA that may aggravate disease severity. Based on the developmental characteristics of the chicken oviduct, 14-day-old layer chicks were used to evaluate oviduct lesions in this study. At this stage, the oviduct remains in an early phase of development, during which epithelial cell proliferation has only just begun. Structures such as oviductal glands, mucosal folds, and tubular glands have not yet been formed, and the oviduct has not entered the critical period of morphogenesis and functional differentiation. In normal layer chickens, oviductal epithelial cells proliferate slowly between 8 and 14 weeks of age, and no significant increase in oviduct length is observed during this period. Oviductal mucosal folds generally do not begin to develop until after 15 weeks of age. Between 18 and 20 weeks of age, the oviduct enters a period of rapid development regulated by estradiol, during which complete glandular and folded structures are gradually formed. Thus, the oviducts of 14-day-old chicks lack the glandular secretions and structural basis required for caseous plug formation. Although IBV can invade the reproductive tract epithelium and potentially affect oviduct development, its pathogenicity toward immature reproductive tract epithelium appeared to be relatively weak in this study and was insufficient to induce gross oviduct lesions [28,29]. This may explain why IBV single infection did not cause oviduct obstruction in this study. This finding contrasts sharply with the pathological characteristics of RA infection, which can directly induce caseous obstruction. These observations further substantiate the conclusion that RA is the main pathogen associated with oviduct obstruction in layer chicks under the experimental conditions used in this study.

Co-infection with IBV and RA exacerbated lesion severity and increased RA associated mortality, indicating that IBV infection may aggravate the clinical outcome of subsequent or concurrent RA infection. The tracheal ciliary stasis assessment in the single infection experiment suggested that this synergism may be related to IBV induced damage to the respiratory ciliated epithelium. IBV infection can cause ciliary stasis, epithelial cell shedding, and abnormal mucus secretion, thereby impairing mucociliary clearance and weakening the mucosal barrier, which may facilitate bacterial adhesion, colonization, and invasion [30]. These findings may contribute to understanding why RA associated co-infections can lead to more severe clinical outcomes in chickens, especially under conditions where respiratory pathogens are circulating. In addition, viral infection-induced immunosuppression is often associated with secondary bacterial infection. Similar phenomena have been reported in IBV-related secondary bacterial infections, particularly those involving *Escherichia coli*. Xiang et al. [31] established an infection model in layer chicks and demonstrated that prior IBV infection significantly enhanced *E. coli* adhesion and colonization, increased bacterial loads in target tissues, and aggravated inflammatory lesions. These observations support the hypothesis that IBV predisposes chickens to secondary bacterial infection by disrupting epithelial integrity and impairing local immune defenses.

In the present study, similar mechanisms may have contributed to the more severe disease phenotype observed in the co-infection group. However, because pathogen burden, immune-response markers, and epithelial barrier-related indicators were not measured, this interpretation should be regarded as a hypothesis rather than a demonstrated mechanism. Future studies should determine whether IBV infection increases RA bacterial loads in the trachea, lungs, air sacs, blood, liver, pericardium, and oviduct, and whether such changes correlate with lesion severity and mortality [32,33]. It is also noteworthy that the differing inoculation routes used in our model aerosolization for RA and oculonasal drop for IBV may influence the initial dynamics of pathogen distribution and subsequent disease development. As supported by Ratanasethakul and Cumming [34], oculonasal and aerosol IBV inoculation only generate minor temporal differences in tracheal lesions, whereas the core phenotype of respiratory mucosal damage, renal pathological changes and systemic viral spread remains consistent regardless of delivery method. While the oculonasal route primarily targets the upper respiratory tract and conjunctiva to establish initial viral replication and mucosal damage, aerosolization allows the bacteria to deposit deeply and uniformly into the lower respiratory tract, including the lungs and air sacs. Thus, virus-induced impairment of upper airway clearance mechanisms may have contributed to the increased severity of respiratory disease following bacterial challenge. However, because tissue-specific loads of RA and IBV were not quantified, it remains unclear whether and how the inoculation route affected pathogen distribution. Therefore, further studies are needed to elucidate the molecular mechanisms underlying this synergistic pathogenesis.

This study found that 7-day-old chicks were significantly more susceptible to infection than their 14-day-old counterparts. This age-dependent susceptibility mirrors the pattern observed in ducks infected with duck-derived RA [35],where ducklings experience severe morbidity and mortality, while adult ducks typically develop subclinical or asymptomatic infections. In light of their developmental characteristics, the incomplete maturation of the oviduct in young chicks, alongside their underdeveloped immune functions and mucosal barriers, likely renders them highly vulnerable to pathogen invasion and subsequent synergistic damage. As birds age, the immune systems of the respiratory and reproductive tracts gradually mature, thereby enhancing resistance to co-infections. Consequently, it can be hypothesized that age-related differences in susceptibility are intrinsically linked not only to host immune competence but also to the developmental stage of the oviduct, the maturity of the mucosal barrier, and the patterns of pathogen synergism. Clinically, IBV, a common respiratory virus, frequently predisposes the host to secondary bacterial infections, including RA. Young layer chickens thus represent a highly vulnerable population to these co-infections, which typically manifest with clinical signs such as renal enlargement and respiratory distress [36].

Building upon the current findings, future studies should quantify post-infection serum antibody levels, cytokines, and local mucosal immunity indicators to elucidate host immune response dynamics during the synergistic pathogenesis of IBV and RA. Additionally, evaluating pathogen loads in target tissues across different infection groups is essential to determine how IBV co-infection impacts the colonization, proliferation, and dissemination of RA within the respiratory tract and oviduct. Mechanistically, in-depth molecular studies are warranted to clarify how IBV disrupts the respiratory epithelial barrier and modulates inflammatory pathways.

In summary, this study clarified the pathogenic characteristics of RA infection and the aggravated disease phenotype associated with IBV and RA co-infection in layer chicks. The results indicate that chicken-derived RA is the primary pathogen responsible for salpingitis in these flocks, whereas IBV may aggravate RA-associated disease, potentially through damage to the respiratory mucosa and impairment of local defense mechanisms. Younger birds exhibited higher susceptibility. While our gross pathological and clinical findings strongly suggest an aggravating effect of IBV on RA infection, the exact synergistic mechanisms require further elucidation. Furthermore, the relatively small group sizes in our in vivo trials may limit the statistical robustness of mortality and lesion incidence comparisons. Despite these limitations, these findings have significant clinical and theoretical value, providing important scientific evidence for the precise diagnosis, prevention, and control of such complex infectious diseases.

## 5. Conclusions

This study shows that serotype 10 chicken-derived RA is a major pathogen associated with fibrinous pericarditis, airsacculitis, perihepatitis, and caseous oviduct occlusion in layer chicks under the experimental conditions used in this study. Solitary infection with the GI-19 lineage of IBV induced severe tracheal ciliary injury but did not cause caseous plug formation in the oviduct. Compared with RA infection alone, IBV pre-infection was associated with more severe clinical signs, higher mortality, and increased lesion incidence, suggesting that IBV may aggravate RA-associated disease outcomes. This aggravating effect may be related to IBV-mediated impairment of the respiratory mucosal barrier; however, RA colonization, IBV replication, tissue distribution, and host immune responses were not quantified in this study, and therefore the underlying mechanism requires further investigation. An age-dependent susceptibility was also observed, with 7-day-old chicks being more vulnerable than their 14-day-old counterparts. Collectively, these findings support the role of RA as an important pathogen involved in this oviduct disease and indicate that IBV may act as a potential aggravating factor in RA-associated disease, providing useful experimental evidence for the prevention and management of RA and IBV co-infections in layer chicks.

## Figures and Tables

**Table 1:** Grouping and challenge methods for the single-infection experiment.

Group	Number	Pathogen	Dose	Route
D1	20	RA	1 × 10^9^ CFU	Aerosol
D2	20	IBV	10^4.5^EID_50_/0.2 mL	Oculo-nasal
D3	20	PBS	0.2 mL	Oculo-nasal

**Table 2:** Grouping and challenge methods of co-infection experiment.

Group	Number	Pathogen	Dose	Route
H1	10 7-day-old layer chicks	RA	1 × 10^9^ CFU	Aerosol
	10 14-day-old layer chicks			
H2	10 7-day-old layer chicks	IBV + RA	IBV: 10^4.5^ EID_50_/0.2 mL RA: 1 × 10^9^ CFU	IBV: Oculo-nasal RA: Aerosol
	10 14-day-old layer chicks			
H3	10 7-day-old layer chicks	PBS	0.2 mL	Oculo-nasal
	10 14-day-old layer chicks			

**Table 3:** Summary of pathogenicity test results for single infection.

Groups	7 dpi				14 dpi				21 dpi			
	Pericarditis	Airsacculitis	Perihepatitis	Fallopian Tube Obstruction	Pericarditis	Airsacculitis	Perihepatitis	Fallopian Tube Obstruction	Pericarditis	Airsacculitis	Perihepatitis	Fallopian Tube Obstruction
D1	4/5	5/5	3/5	5/5	3/5	4/5	1/5	5/5	3/5	3/5	2/5	3/5
D2	0/5	0/5	0/5	0/5	0/5	0/5	0/5	0/5	0/5	0/5	0/5	0/5
D3	0/5	0/5	0/5	0/5	0/5	0/5	0/5	0/5	0/5	0/5	0/5	0/5

Abbreviations: dpi, days post-infection.

**Table 4:** Summary of pathogenicity test results for co-infection.

Groups	7-Day-Old					14-Day-Old				
	Pericarditis	Airsacculitis	Perihepatitis	Fallopian Tube Obstruction	Death	Pericarditis	Airsacculitis	Perihepatitis	Fallopian Tube Obstruction	Death
H1	5/10	2/10	5/10	2/10	0/10	4/10	2/10	2/10	1/10	0/10
H2	6/7	4/7	6/7	4/7	3/10	6/8	6/8	6/8	4/8	2/10
H3	0/10	0/10	0/10	0/10	0/10	0/10	0/10	0/10	0/10	0/10

## Data Availability

The raw data supporting the findings of this study are available from the corresponding author upon reasonable request.
